# Burden of death associated with SARS-CoV-2 infection during the pandemic in Flint, Michigan (MI), mortality trends over the 2-year period: impact of social and health inequities

**DOI:** 10.1017/ash.2024.346

**Published:** 2024-08-07

**Authors:** Mariam Younas, Danielle Osterholzer, Carlos F. Ríos-Bedoya

**Affiliations:** 1 Department of Medicine, Hurley Medical Center, Flint, MI, USA; 2 College of Human Medicine, Department of Medicine, Michigan State University, East Lansing, MI, USA; 3 College of Human Medicine, Department of Epidemiology and Biostatistics, Michigan State University, East Lansing, MI, USA; 4 McLaren Health Care, Graduate Medical Education, Grand Blanc, MI, USA

## Abstract

**Background:**

This cross-sectional study aims to determine the mortality trends in patients with SARS-CoV-2 infection during the pandemic in Flint, MI.

**Methods:**

Records from 1,663 consecutive adult patients (≥18 years of age) with confirmed SARS-CoV-2 infection, admitted and discharged from our facility from 03/2020 through 02/2022, were abstracted and analyzed. Multivariable logistic regression analysis was performed to examine the association between study explanatory variables (ie, sex, age, co-morbidities, etc.) and the primary study outcome (ie, mortality).

**Results:**

During the 2-year study period, the overall crude 90-day mortality rate was 16.1% (267/1663), being lowest in the period 5 (Table 1). Male sex, older age, certain co-morbidities, supplemental oxygenation use, and lack of immunization were associated with mortality. Therapeutics such as remdesivir and steroids were not associated with improved survival.

**Conclusion:**

Despite substantial changes in supportive care, management and circulating variants, SARS-CoV-2 carried a significant mortality risk. Vaccination coverage in this high-risk study sample was low, at only 12%. Public health efforts should be focused at overcoming the barriers to vaccine acceptance in this high-risk unique population.

## Introduction

Approximately 3.2 million confirmed and probable cases have been reported in the state, causing 43,830 deaths.^1^ With the onset of pandemic, COVID-19 disproportionally affected racial and ethnic minority groups and many survivors continue to experience long-term sequelae of infection adding to the health and socioeconomic burden of these already strained populations.^2^ Communities like Flint, MI faced an increased vulnerability to SARS-CoV-2 infection with much higher mortality and complications compared to other communities in MI.^1^ MI statewide case fatality rate is 1.4%, for Genesee County (where Flint is located) it is 1.9%. More affluent counties such as Washtenaw and Oakland County are 0.7%, and 1.2% respectively.^1^

The purpose of this cross-sectional study is to describe our experience with COVID-19 infection in Flint, MI and determine factors associated with higher mortality amongst those hospitalized in this low-income, underserved community. There is lack of studies examining and comparing the first and subsequent COVID-19 waves, not just in Flint but in MI as well. To our knowledge, this is the largest and only observational study to date from Flint, MI comparing the first and subsequent COVID-19 waves amongst a large racially and ethnically diverse population.

## Methods

This study was approved by the institutional review board of Hurley Medical Center-Michigan State University College of Human Medicine. An informed consent was waived as this study conducted a chart data abstraction and the patients were not subjected to additional testing.

### Data source and study population

This study was conducted at Hurley Medical Center (HMC), Flint MI. Hurley is a 443-bed premier public teaching hospital (largest in the region) recognized as a regional leader in advanced specialized health care and is the region’s only Level 1 Trauma Center, and has the region’s only Burn Unit, Level III Neonatal Intensive Care Unit, Pediatric Intensive Care Unit, and Pediatric Emergency Department.

We retrospectively collected basic demographic, laboratory, radiologic, use of supplemental oxygen and specific treatment data, as well as outcomes on adults (≥18 years {yrs} of age) with lab confirmed SARS-CoV-2 infection (using nucleic acid amplification test and antigen-based testing), admitted and discharged from our facility from March 2020 through February 2022. All individuals with lab confirmed SARS-CoV-2 infection, including cases found incidentally, were included in this study. The 2-year span was divided into six periods coinciding with the six consecutive epidemiological waves of COVID-19 infection in MI (Table 1). A total of 16,163 patients were tested for COVID-19 infection and 2,860 positive cases with 1,663 unique patients requiring hospitalization (including 6 recurrent infections) were seen through the Emergency Department during the study time frame. Repeat testing within 3 months of an initial test was not allowed at our institution.

The primary study outcome were the mortality trends (90-day mortality from the first positive test) in patients hospitalized with COVID-19 infection during the 2-year study time frame. Secondary outcomes were the length of stay (LOS), use of supplemental oxygenation, need for invasive mechanical ventilation (MV), and association of available therapeutics and immunization against COVID-19 infection related mortality as encountered during the index hospitalization for incident and recurrent cases. Age, sex, race, co-morbidities, immunization and use of COVID-19 therapeutics were considered as study explanatory/clinical variables.

### Statistical analysis

Descriptive statistics including mean, standard deviation (SD), and proportions were used to summarize the data, pertaining to patient demographics such as age, gender, race and prevalence of pre-existing conditions. Multivariable logistic regression analysis was performed to examine the association between study explanatory variables (ie, sex, age, co-morbidities, etc.) and the study outcome (ie, mortality) and reported as adjusted odds ratios along with 95% confidence intervals. Descriptive statistics were used to define the mortality trends during the six consecutive waves and the population at risk.

The usual alpha-level of 0.05 was used to determine statistical significance and all statistical tests were two-sided. All data was analyzed using STATA version 11.

## Results

Patient demographic characteristics and co-morbidities as seen during the six consecutive epidemiological waves of COVID-19 infection in MI are presented in Table 1. The mean age of the cohort was 58.6 yrs (SD ±18) and there was a slight trend toward a younger population near the end of the study period (Table 1). African Americans (AA) comprised 50.9% (831/1631) and whites 43.9% (717/1631) of the study sample. There was a shift in demographics across the 2 years with AA being the predominantly infected patients initially to a more homogenous racial mix toward the end of the study (Table 1). Women comprised 49.9% of the cohort (830/1663). Mean Body Mass Index (BMI) was 31.7 (SD ±9.6). Hypertension was the most common co-morbidity, 61% of the study sample suffered from it. During the study period, 12.5% of the cohort (207/1663) had received at least one dose of available COVID-19 vaccines (including single dose of Johnson and Johnson’s Jansen vaccine).

**Table 1.** Characteristics of individuals with SARS-CoV-2 infection during the first and subsequent waves of the pandemic, immunization against COVID-19 and use of COVID-19 therapeutics in Flint, MI

	1^st^ wave	2^nd^ wave	3^rd^ wave	4^th^ wave	5^th^ wave	6^th^ wave
Age in years, mean ± SD	60.9 ± 16.6	61.2 ± 17.03	59.3 ± 18.5	57.5 ± 17.7	55.5 ± 18.1	58.3 ± 18.2
Race, no. /total no. (%)						
AA	177/226 (78.3)	31/74 (41.9)	177/319 (55.5)	136/255 (53.3)	63/184 (34.2)	247/573 (43.3)
White	41/226 (18.1)	33/74 (44.6)	128/319 (40.1)	110/255 (43.1)	111/184 (60.3)	294/573 (51.3)
Female, no. /total no. (%)	109/229 (47.6)	38/75 (50.7)	163/322 (50.6)	127/259 (49.0)	96/187 (51.3)	297/591 (50.3)
BMI (kg/m^2^) ± SD	31.3 ± 8.4	33.0 ± 9.5	32.3 ± 10.1	32.6 ± 9.7	32.2 ± 10.1	30.8 ± 9.7
Co-morbidities, no./total no. (%)						
HTN	160/229 (69.8)	45/75 (60)	206/322 (63.9)	152/259 (58.7)	95/187 (50.8)	355/591 (60.1)
DM	88/229 (38.4)	28/75 (37.3)	118/322 (36.6)	85/259 (32.8)	55/187 (29.4)	188/591 (31.8)
COPD	26/229 (11.4)	15/75 (20)	49/322 (15.2)	38/259 (14.7)	29/187 (15.5)	91/591 (15.4)
CKD	33/229 (14.4)	8/75 (10.7)	28/322 (8.7)	32/259 (12.4)	10/187 (5.3)	70/591 (11.8)
ESRD	23/229 (10)	4/75 (5.3)	14/322 (4.3)	18/259 (6.9)	4/187 (2.1)	43/591 (7.3)
CAD	12/229 (5.2)	2/75 (2.7)	21/322 (6.5)	14/259 (5.4)	5/187 (2.7)	33/591 (5.6)
CLD	38/229 (16.6)	16/75 (21.3)	66/322 (20.5)	43/259 (16.6)	36/187 (19.3)	112/591 (18.9)
Immunization, no. /total no. (%)						
AA	0	0	0	2/128 (11.0)	13/63 (20.6)	56/247 (22.7)
White	0	0	2/128 (1.6)	10/110 (9.1)	18/111 (16.2)	83/294 (28.2)
RDV use, no./total no. (%)						
AA	5/177 (2.8)	19/31 (61.3)	92/177 (51.9)	85/136 (62.5)	45/63 (71.4)	140/247 (56.7)
White	0	21/33 (63.6)	66/128 (51.6)	74/110 (67.3)	75/111 (67.6)	161/294 (54.8)
mAB use, no./total no. (%)						
AA	0	0	2/177 (1.1)	9/136 (6.6)	0	32/247 (12.9)
White	1/41 (2.4)	0	1/128 (0.8)	7/110 (6.4)	0	20/294 (6.8)

Note. AA, African American; BMI, Mean Body Mass Index; SD, standard deviation; ESRD, end stage renal disease; COPD, chronic pulmonary obstructive disease; CAD, coronary artery disease; CKD, chronic kidney disease; DM, diabetes; HTN, hypertension; CLD, chronic liver disease; RDV, Remdesivir; mAB, monoclonal antibody.

1^st^ wave – March 2020 – June 2020.

2^nd^ wave – July 2020 – October 2020.

3^rd^ wave – Nov 2020 – Feb 2021.

4^th^ wave – March 2021 – June 2021.

5^th^ wave – July 2021 – October 2021.

6^th^ wave – Nov 2021 – Feb 2022.

**Figure 1.**
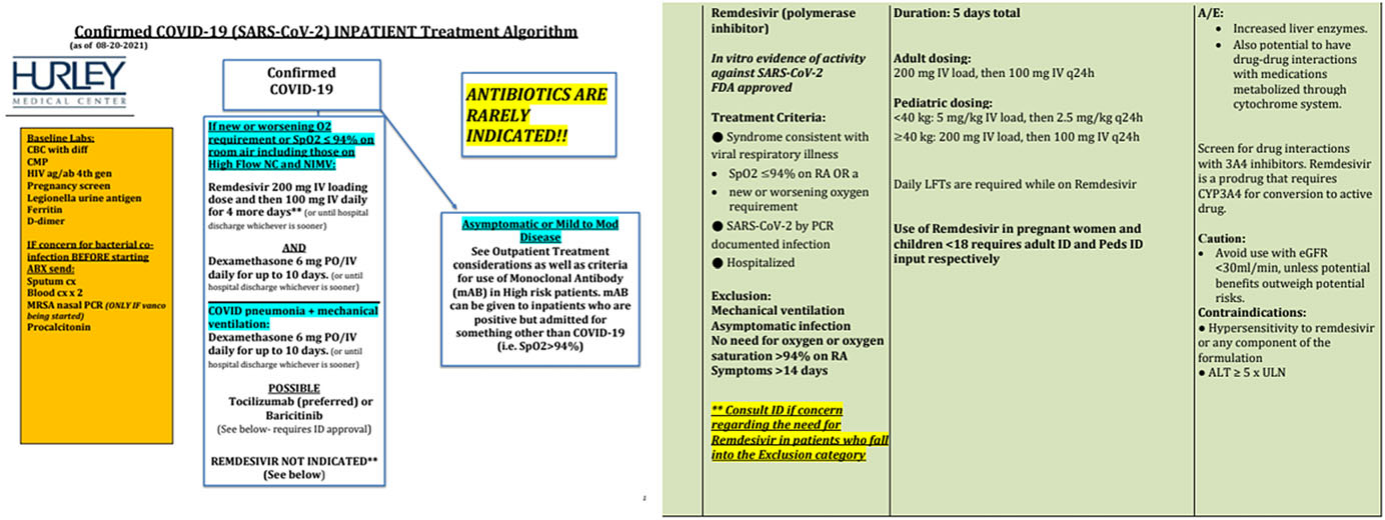
Institutional protocol.

At the onset of the pandemic, 158 patients received hydroxychloroquine (HQC) as a potential treatment for COVID-19, 144 of them receiving in first wave. This drug was abandoned as it appeared ineffective over time and as remdesivir (RDV) became the standard of care. The first dose of RDV was administered at HMC on 05/13/2020. Based on the institutional protocol, 858/1663 (51.6%) received RDV, 1066/1663 (64.1%) received steroids and 72/1663 (4.3%) hospitalized patients received monoclonal antibody (mAb) (Fig. 1). The mAb treatment was only administered to those patients who didn’t require oxygen and were determined to have mild COVID-19 infection. The type of mAb administered depended on the local, national guidelines and COVID-19 circulating strain at that time period (Table 2).

**Table 2.** Type of monoclonal antibodies administered – time line at Hurley

Bamlanivimab monotherapy administered from 11-19-20 to 4-16-21.
Bamlanivimab-Etesevimab administered from 4-16-21 to 6-16-21 and 11-17-21 to 1-20-22.
Casirivimab-Imdevimab IV administered from 7-8-21 to 12-23-21.
Casirivimab-Imdevimab s/c administered from 12-2-21 to 1-19-22.
Sotrovimab administered from 12-2-21 to 3-31-22.
Bebtelovimab administered from 4-4-22 to 11-30-22.

### COVID-19 outcomes

During the 2-year study period, the overall crude 90-day mortality rate in this hospitalized cohort was 16.1% (267/1663) and the In-hospital case fatality rate was 14.1% (235/1663). 90-day mortality was lowest in wave 5 (Table 3). Non-survivors tended to be older, mean age of 68 vs 57 yrs (*P* < 0.001). The mean LOS was 9.8 days (SD ±15.2), longer in non-survivors, 14.9 days (SD ±13.9) vs 8.9 days (SD ±15.2, *P* < 0.001) in survivors. The crude mortality in AA (16.3%, 135/831) and whites (15.2%, 109/717) remained relatively similar throughout the waves (Table 3). After adjusting for age, sex, and BMI, AA did not have higher odds of dying compared to their white peers (adjusted Odds Ratio for mortality [adj. OR] 1.1, 95% confidence interval [CI] 0.86–1.53, *P* = 0.360). A total of 157 (58.8% of the non-survivors) men died compared to 110 women (41.2% of the non-survivors) during the study period. Multivariate logistic regression showed that male sex was associated with a higher odd of dying (adj. OR 1.8, 95% CI 1.32–2.37, *P* < 0.001).

**Table 3.** Comparative mortality based on race and predominant SARS-CoV-2 circulating strain

	1^st^ wave	2^nd^ wave	3^rd^ wave	4^th^ wave	5^th^ wave	6^th^ wave
Crude mortality, no./total no. (%)	55/229 (24.0)	14/75 (18.7)	52/322 (16.2)	44/259 (16.9)	24/187(12.8)	78/591 (13.2)
AA	42/177 (23.7)	5/31 (16.1)	25/177 (14.1)	22/136 (16.2)	8/63 (12.7)	33/247 (13.4)
White	11/41 (26.8)	6/33 (18.2)	25/128 (19.5)	19/110 (17.3)	13/111(11.7)	35/294 (11.9)
Predominant SARS-CoV-2 Strain/Variants of concern in US	SARS-CoV-2 GH strain	SARS-CoV-2 GH strain	Alpha (B.1.1.7 lineage)	Alpha	Delta (B.1.617.2)	Delta (Nov, Dec)-Omicron (B1.1.529) (Dec-onwards)

*1st wave – March 2020 – June 2020, 2^nd^ wave – July 2020-October 2020, 3^rd^ wave – Nov 2020-Feb 2021, 4^th^ wave – March 2021 – June 2021, 5^th^ wave – July 2021-October 2021, 6^th^ wave – Nov 2021 – Feb 2022.

Hypertension, Chronic Obstructive Pulmonary Disease (COPD) and Diabetes Mellitus was significantly associated with mortality after adjusting for age, race, sex, and BMI (Table 4). Immunization against COVID-19 was associated with a lower risk of mortality (adj. OR 0.54, 95% CI 0.34–0.85, *P* = 0.008, adjusted for age, race, sex, BMI).

**Table 4.** Co-morbidities and outcomes

Co-morbidities	
HTN, no. /total no. (%)	1013/1663 (60.9)
AA	590/831 (71.0)
White	362/717 (50.5)
Crude mortality	190/267 (71.2)
Adj. OR (95% CI)	1.7 (1.27–2.25)
DM, no. /total no. (%)	562/1663 (33.8)
AA	312/831 (37.6)
White	210/717 (29.3)
Crude mortality	108/267 (40.4)
Adj. OR (95% CI)	1.4 (1.07–1.83)
COPD, no. /total no. (%)	248/1663 (14.9)
AA	123/831 (14.8)
White	116/717 (16.2)
Crude mortality	56/267 (20.9)
Adj. OR (95% CI)	1.7 (1.19–2.31)
CKD, no. /total no. (%)	181/1663 (10.9)
AA	117/831 (14.1)
White	55/717 (7.7)
Crude mortality	40/267 (14.9)
Adj. OR (95% CI)	1.6 (1.07–2.28)
ESRD, no. /total no. (%)	106/1663 (6.4)
AA	69/831 (8.3)
White	30/717 (4.2)
Crude mortality	21/267 (7.9)
Adj. OR (95% CI)	1.3 (0.79–2.15)
CAD, no. /total no. (%)	87/1663 (5.2)
AA	39/831 (4.7)
White	41/717 (5.7)
Crude mortality	21/267 (7.9)
Adj. OR (95% CI)	1.7 (1.03–2.85)
CLD, no. /total no. (%)	311/1663 (18.7)
AA	155/831 (18.7)
White	143/717 (19.9)
Crude mortality	64/267 (23.9)
Adj. OR (95% CI)	1.5 (1.07–1.99)

Note. ESRD, end stage renal disease; COPD, chronic pulmonary obstructive disease; CAD, coronary artery disease; CKD, chronic kidney disease; DM, diabetes; HTN, hypertension; CLD, chronic liver disease; 95% CI, 95% confidence interval; Crude mortality, non-survivors with co-morbidity/total no. of non-survivors; adj. OR, adjusted Odds Ratio.

Overall, 1,217 patients received supplemental oxygenation including 227 needing non-invasive ventilation (BiPaP), and 376 needing MV. Some patients who were initially on BiPaP eventually progressed to needing MV. A total of 376/1663 (22.6%) patients required mechanical ventilatory support. While 329/376 (87.5%) and 263 /376 (69.9%) of these patients received steroids and RDV respectively. All the ventilated patients who received RDV (263) were also on steroids. Use of supplemental oxygenation and MV were independent risk factors predictive of higher risk of mortality {adj (age, race, sex, BMI) OR 2.7, 95% CI 1.57–4.71, *P* < 0.001 and OR 24.0, 95% CI 14.02–41.25, *P* < 0.001, respectively}.

After adjusting for age, race, sex, BMI and co-morbidities, HQC was associated with higher odds of mortality (adj. OR 1.7, 95% CI 1.14–2.59, *P* = 0.01). RDV use was associated with a higher risk of mortality (adj. OR 1.5, 95% CI 1.15–1.97, *P* = 0.003, adjusted for age, race, sex, BMI, co-morbidities), with no survival benefit in mechanically ventilated group (adj. OR 0.9, 95% CI 0.64–1.55, *P* > 0.999) or those requiring supplemental oxygenation (adj. OR 1.0, 95% CI 0.78–1.39, *P* = 0.780).

Steroid use also was associated with a higher risk of mortality {adj. OR for mortality (adjusting for age, race, sex, BMI) 2.9, 95% CI 2.08–4.01, *P* <0.001 and after adjusting for the above-mentioned variables and use of supplemental oxygenation OR 2.0, 95% CI 1.39–2.97, *P* <0.001}. The combination of RDV and steroids failed to show a survival benefit as well {adj. (age, race, sex, BMI, COPD, immunization, oxygen use) OR 2.6, 95% CI 1.84–3.62, *P* <0.001}.

There were six deaths reported in the mAb group (6/72). Use of mAb was associated with a lower risk of mortality {adj OR (age, race, sex, BMI, immunization) 0.46, 95% CI 0.19–1.07, *P* = 0.07}.

### COVID-19 infection and disparities in care

We also tried to assess if disparities existed in RDV and mAb receipt between the two major ethnic groups along with differences in COVID-19 immunization rates and existing co-morbidities. Utilization of mAB therapy was consistent among the AA and whites, except in period 6 where 12.8% of AA received mAb compared to 6.6% of the whites. Overall, 5% (43/831) of AA received mAb compared to 4% (29/717) whites (60% of those who received mAb were AA and 40% were white). Overall, 55.4% (397/717) of whites’ vs 46.4% (386/831) of AA received RDV. AA as a group had more co-morbidities compared to whites (Table 4). Crude vaccination coverage rate was delayed in AA and was slightly behind their white peers (Table 1). 15.8 % (113/177) of whites compared to 10.1% (84/831) (*P* < 0.001) of AA received an immunization against COVID-19 infection. There was a disparity identified in RDV utilization among the two races despite the fact that AA had two or more co-morbidities compared to their white peers (388 vs 260). Use of supplemental oxygenation was similar in both groups (72% – AA 599/831, whites 517/717). More AA required mechanical ventilation compared to their white peers {22.9% (190/831) vs 20.8% (149/717)}.

## Discussion

This high-risk group of hospitalized COVID-19 infected patients in Flint, Michigan had a 90-day mortality rate of 16.1% and an in-hospital mortality rate of 14.1%. Despite the population dynamics of the community, our study sample was racially homogenous (AA comprised 51% and whites 44%). Michigan is among the states with the highest number of COVID-19 cases and deaths.^1,3^ According to U.S. Census Bureau data, Genesee County’s current population of 404,208 includes a racial composition of 75.0% white, 20.3% AA, and 3.9% Hispanic/Latino.^4^ Nearly 200,000 people lived within the city of Flint during its peak in the 1960s–1970s, today only 80,628 residents remain, a majority being AA (56.7%).^4,5^ Socioeconomic and environmental factors influence the health status among residents throughout Genesee County, with significant impact on residents of Flint. The poverty rates for both Genesee County (16.3%) and the city of Flint (35.5%) are much higher than state (13.1%) and national (11.6%) rates. High school graduation rates for students in Genesee County consistently fall slightly below the statewide average, while those for Flint Community School students are considerably lower at 52% compared to the statewide average of 82%.^5,6^ Genesee County’s obesity rate is significantly higher than state and national averages, and the combined obesity and overweight rate for Genesee County is 72.4%.^4,6,7^ In terms of environmental factors, Flint is still recovering from the well-known Flint water crisis. The City of Flint *Fast Start Program*, with a goal of replacing all lead service lines in the city, was scheduled to be completed by 2020, however, as of September 2022 that process is still ongoing.^8^

The many socioeconomic and environmental disparities that the Flint and adjoining community face, has led to poor and delayed access to health care (lack of easy healthcare access including insurance coverage, transportation, ability to do televisits, especially important with the stay at home and social distancing guidelines during the pandemic), lack of early testing for COVID-19 infection, not recognizing warning signs/symptoms on time, lack of knowledge of preventative therapies and being skeptical and distrustful of governmental guidance, and hence more prone to misinformation, eventually leading to poorer health outcomes and behavior. Based on our study and data from Michigan Department of Health and Human Services-Genesee County and the City of Flint has the highest overall COVID-19 related mortality.^1^ Although in the beginning of the pandemic, AA were perceived to be at higher risk of death, our results indicated otherwise, showing that despite having more co-morbidities, the crude mortality was similar in both races. A multivariate analysis failed to show association of mortality to race.^9–11^ It is plausible that the higher mortality seen in minorities at the beginning of the pandemic was related to the higher risk of exposure and contracting the infection, which eventually translated to higher number of cases in minorities and higher deaths.^12^ A cohort study of approx. 11,000 patients with COVID-19 infection presenting for care at 92 hospitals across 12 US states, failed to show a difference in all-cause, in-hospital mortality between white and AA patients after adjusting for age, sex, insurance status, co-morbidity, neighborhood deprivation, and site of care.^13^ We did see a difference in provision of care once hospitalized for AA vs white patients as suggested by a prior study.^14^ RDV use was slightly more in whites compared to AA and this difference could not be solely explained by the difference in need for mechanical ventilation on presentation. Potential confounders such as presence of a contraindication to administration of RDV on presentation of the patient (such as elevated transaminases, and/or presence of severe renal failure), presenting with over 2 weeks of symptoms or patient/guardian refusing administration of RDV, could account for the difference in RDV utilization in between the two races, though their true effect was not studied.

COVID-19 related mortality did improve over the course of 2 years as the pandemic progressed. However, it remained significant regardless of the circulating variant.^15^ Our multivariate analysis failed to show a mortality benefit with therapeutics such as RDV or steroids likely in part due to delayed receipt of these medications when the disease was already severe. This study adds to the growing evidence that more efficacious treatments against COVID-19 respiratory failure are needed once a patient is hospitalized and that antiviral therapies at this late stage may not have the desired effect.^16–18^ On the other hand, use of mAB and immunization against COVID-19 appeared to be associated with improved mortality though our patient population who received them were small in number. Optimizing these resources in this high-risk population is key to reducing unfavorable outcomes.^19–22^

The current study has several limitations, the biggest being the observational cross-sectional design with no model for randomization. Hospitalized patients were likely sicker and the standard of care therapies based on institutional and national guidelines failed to show a mortality benefit once respiratory failure set in. Studies have shown benefit to using RDV and other antivirals such as nirmatrelvir with ritonavir (Paxlovid) early in the course of the illness in high-risk patients.^23,24^ In this study, some of the patients who had COVID-19 infection and were hospitalized may have died of other causes. Particularly in later stages of the study, COVID-19 infection may not have been the primary cause of admission, and the outcome differences could be slightly over- or under-estimated. Variables under study, including race were extracted from the Electronic Medical Record and were not confirmed by interview with the patients. We did not assess the duration of steroid use. Some patients were started on methylprednisolone or hydrocortisone and transitioned to dexamethasone or vice versa. There was a tendency toward longer than 10-day courses of steroids, when patients failed to improve or remained in respiratory failure. The effect of this on patient outcomes is unclear and we did not attempt to address this. We did not capture all hospitalized patients with COVID-19 infection in the Flint area as some of the COVID-19 patients received care at the other area hospitals. Therefore, extrapolating the results or mortality analysis to the entire community could be a slightly under- or over-estimation of the current problem. However, this in-hospital-based approach is likely a good risk assessment model based on the study design. Also with this study design, we could not fully assess the reason behind the difference in RDV utilization that was seen among the two major racial groups. Lastly use of social distancing guidelines and adherence to masking as a potential cause of reduced infection rate in AA during the pandemic could not be assessed with the current study design. The cross-sectional nature of the study design limits the ability for establishing the temporal sequence of events. However, since COVID-19 is an infectious disease, most of the patient level factors and co-morbidities were already present at the time of infection. As with all observational studies, there is always a possibility that an unmeasured confounder could have a large impact on our outcome. Nonetheless, given our current knowledge of COVID-19, the chances of this happening in the present study are very low.

Notwithstanding these limitations, our study also has several strengths. This single center observational study assessed and compared the outcomes of COVID-19 infection and available therapeutics and provides a glimpse of real-world experience in a racially homogenous group. It furthers our understanding of population dynamics and their interplay with diseases and predictors of poor outcomes. It highlights the importance of prevention, delivery of credible equitable resource such as vaccination and building trusting relationships with the community to improve outcomes.

**Figure 2.**
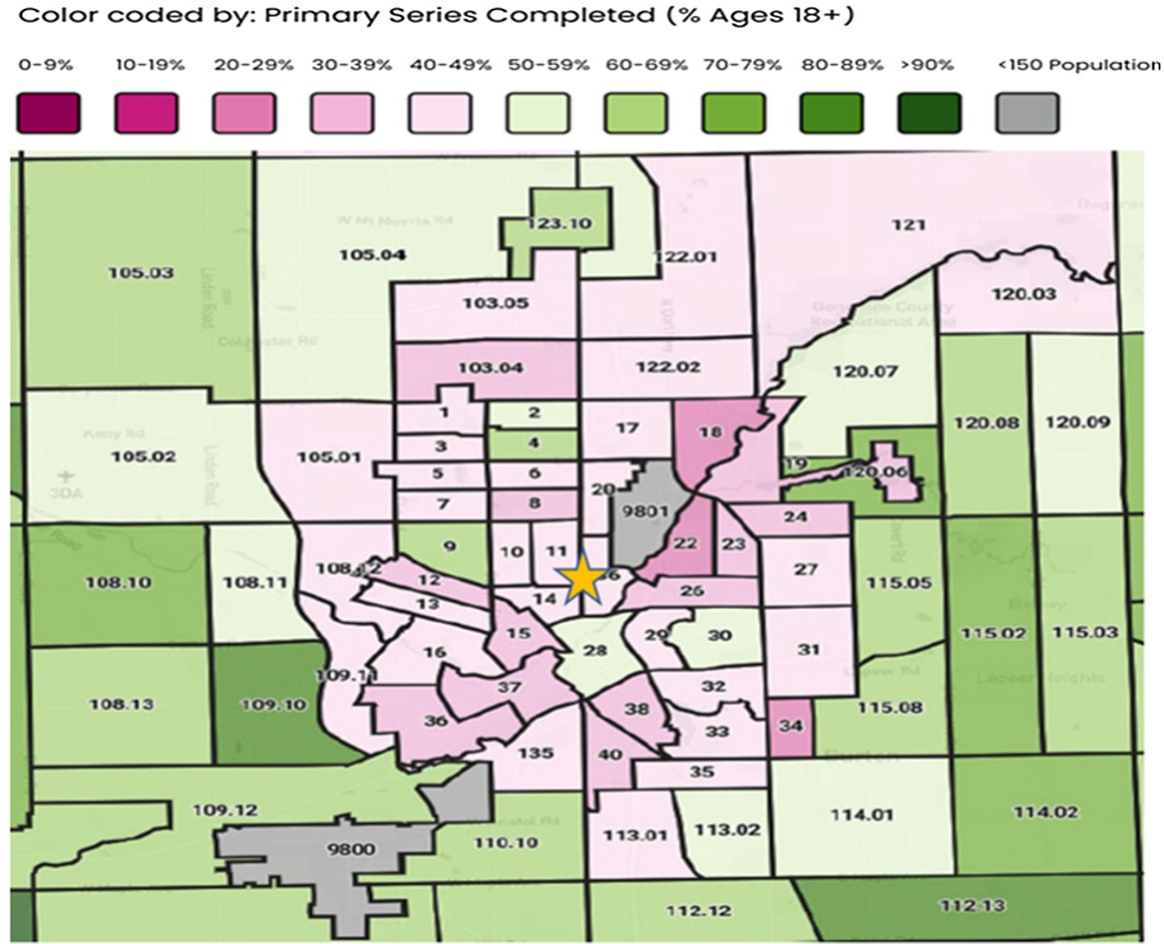
Detailed map of Flint and surrounding areas COVID-19 vaccine status (as of 08/20/2023). Numbers on the maps are the census tracts for each location. The percent population receiving the vaccination is indicated by the legend/color. Yellow star highlights the approximate location of Hurley Medical Center. “Up to Date” refers to individuals who have received the latest recommended dose based on current public health guidance for their age group. www.gchd.us.

In summary, this unique community suffered a disproportionate impact of COVID-19 infection, with severe illness, and death. The many deeply rooted socioeconomic and environmental disparities plaguing this area along with poor vaccine uptake likely contributed to poorer outcomes rather than the provision of care once hospitalized. Vaccination coverage in this high-risk cohort was low, at only 12%. Vaccination coverage in Flint and surrounding areas remains low at less than 50% as reported by the Genesee County health department (Fig. 2). Public health efforts should be focused on overcoming the barriers to vaccine acceptance in this high-risk unique population. We also did not find race as an independent predictor of mortality. Similar outcomes are noted in communities like City of Chicago, which are plagued by similar disparities.^25^

To conclude, and to the best of our knowledge, this is the first and only study from Flint, Michigan, examining the trends of mortality and describing the story of Flint and surrounding community over a period of 2-years as the pandemic unfolded.

## Author Biography

Dr. Younas is an Assistant Professor and a core faculty in the Internal Medicine Residency Program at Hurley Medical Center-Michigan State University/College of Human Medicine and also serve as the Associate Director of the Antimicrobial Stewardship Program and a member of the Pharmacy and Therapeutics Committee (P&T) for the Hurley Medical Center. Before joining Hurley Medical Center, Dr. Younas served as the physician lead of the Antimicrobial Stewardship Collaborative of South Carolina, a collaboration between 50 acute care hospitals, skilled nursing facilities, and ambulatory practices in the state, coordinating antimicrobial stewardship efforts in South Carolina through numerous projects and interventions.

Dr. Younas completed her Internal Medicine residency from Rush University Medical Center, Chicago IL in June 2012 and Infectious Diseases fellowship from Indiana University School of Medicine, Indianapolis IN in August 2015. She served as a Chief Clinical fellow in her final year of Infectious Diseases fellowship.

Her primary research interests include hospital epidemiology, antimicrobial resistance and stewardship.

## References

[1] Michigan data. Available at https://www.michigan.gov/coronavirus/stats. Accessed January 05, 2024.

[2] Hoogenboom WS , Pham A , Anand H , et al. Clinical characteristics of the first and second COVID-19 waves in the Bronx, New York: A retrospective cohort study. Lancet Reg Health Am. 2021;3:100041. doi: 10.1016/j.lana.2021.100041. 34423331 PMC8367084

[3] United States COVID-19 cases and deaths by state over time. Available at https://data.cdc.gov/Case-Surveillance/United-States-COVID-19-Cases-and-Deaths-by-State-o/9mfq-cb36. Accessed February 13, 2023.

[4] U.S. Census Bureau. Available at https://www.census.gov/quickfacts/fact/table/US,MI,flintcitymichigan,geneseecountymichigan/PST045222. Accessed February 13, 2023.

[5] Community health needs assessment report. Available at https://gfhc.org›hc726-CHNA-Report-2022_web. Accessed February 13, 2023.

[6] MI school data. Available at https://www.michigan.gov/cepi/mischooldata. Accessed February 13, 2023.

[7] Behavioral risk factor surveillance system. Available at https://www.cdc.gov/brfss/index.html. Accessed February 13, 2023.

[8] Flint enters final phase of lead service line replacement. Available at https://www.michigan.gov/egle/newsroom/press-releases/2022/09/30/flint-enters-final-phase-of-lead-service-line-replacement. Accessed February 13, 2023.

[9] Ezell JM , Griswold D , Chase EC , et al. The blueprint of disaster: COVID-19, the Flint water crisis, and unequal ecological impacts. Lancet Planet Health. 2021;5:e309–e315. doi: 10.1016/S2542-5196(21)00076-0. 33964240 PMC9709384

[10] Parpia AS , Martinez I , El-Sayed AM , et al. Racial disparities in COVID-19 mortality across Michigan, United States. EClinicalMedicine. 2021;33:100761. doi: 10.1016/j.eclinm.2021.100761. 33718849 PMC7933264

[11] Golestaneh L , Neugarten J , Fisher M , et al. The association of race and COVID-19 mortality. EClinicalMedicine. 2020;25:100455. doi: 10.1016/j.eclinm.2020.100455. 32838233 PMC7361093

[12] Holden TM , Simon MA , Arnold DT , et al. Structural racism and COVID-19 response: higher risk of exposure drives disparate COVID-19 deaths among Black and Hispanic/Latinx residents of Illinois, USA. BMC Public Health. 2022;22:312. doi: 10.1186/s12889-022-12698-9. 35168585 PMC8845334

[13] Yehia BR , Winegar A , Fogel R , et al. Association of race with mortality among patients hospitalized with coronavirus disease 2019 (COVID-19) at 92 US hospitals. JAMA Netw Open. 2020;3:e2018039. doi: 10.1001/jamanetworkopen.2020.18039. 32809033 PMC7435340

[14] Sutton NR , Robinson-Lane SG , Yeow RY , et al. Racial and ethnic variation in COVID-19 care, treatment, and outcomes: A retrospective cohort study from the MiCOVID-19 registry. PLoS One. 2022;17:e0276806. doi: 10.1371/journal.pone.0276806. 36318576 PMC9624408

[15] Younas M , Rios-Bedoya C , Osterholzer D , et al. Burden of death associated with SARS-CoV-2 infection during the pandemic in Flint, Michigan (MI): Mortality trends over the 2-year time period. Open Forum Infect Dis. 2022;9;ofac492.1495. doi: 10.1093/ofid/ofac492.1495. PMC11736443 39823118

[16] Gupta K , Monday L , Kaushik M , et al. Risk factors associated with 30-day mortality in a large cohort of patients who received Remdesivir and corticosteroids for severe COVID-19. Open Forum Infect Dis. 2021; 8:S375–S376, doi: 10.1093/ofid/ofab466.746.

[17] Younas M , Osterholzer D , Rios-Bedoya C , et al. COVID-19 therapeutics: Real-world experience in Flint, Michigan (MI). Open Forum Infect Dis. 2022; 9;ofac492.957, doi: 10.1093/ofid/ofac492.957.

[18] WHO Solidarity Trial Consortium. Remdesivir and three other drugs for hospitalised patients with COVID-19: final results of the WHO Solidarity randomised trial and updated meta-analyses [published correction appears in Lancet. 2022 Oct 29;400(10362):1512]. Lancet. 2022;399:1941–1953. doi: 10.1016/S0140-6736(22)00519-0. 35512728 PMC9060606

[19] Grapsa E , Adamos G , Andrianopoulos I , et al. Association between vaccination status and mortality among intubated patients with COVID-19–related acute respiratory distress syndrome. JAMA Netw Open. 2022;5:e2235219. doi: 10.1001/jamanetworkopen.2022.35219. 36205996 PMC9547321

[20] Johnson AG , Linde L , Ali AR , et al. COVID-19 incidence and mortality among unvaccinated and vaccinated persons aged ≥12 years by receipt of bivalent booster doses and time since vaccination — 24 U.S. jurisdictions, October 3, 2021–December 24, 2022. MMWR Morb Mortal Wkly Rep. 2023;72:145–152. doi: 10.15585/mmwr.mm7206a3. 36757865 PMC9925136

[21] Cohen MJ , Oster Y , Moses AE , et al. Association of receiving a fourth dose of the BNT162b vaccine with SARS-CoV-2 infection among health care workers in Israel. JAMA Netw Open. 2022;5:e2224657. doi: 10.1001/jamanetworkopen.2022.24657. 35917125 PMC9346545

[22] Younas M , Osterholzer D , Flues BR , et al. Monoclonal antibody therapy for COVID-19 infection in Michigan: The Flint experience. Open Forum Infect Dis. 2021;8:S376–S377, doi: 10.1093/ofid/ofab466.748.

[23] Gottlieb RL , Vaca CE , Paredes R , et al. Early Remdesivir to prevent progression to severe Covid-19 in outpatients. N Engl J Med. 2022;386:305–315. doi: 10.1056/NEJMoa2116846. 34937145 PMC8757570

[24] Hammond J , Leister-Tebbe H , Gardner A , et al. Oral Nirmatrelvir for high-risk, nonhospitalized adults with Covid-19. N Engl J Med. 2022;386:1397–1408. doi: 10.1056/NEJMoa2118542. 35172054 PMC8908851

[25] COVID-19 IMPACTS ON LIFE EXPECTANCY IN CHICAGO, 2019-2020. Available at https://www.chicago.gov/city/en/sites/covid-19/home/impact-on-chicago-2020.html. Accessed April 14, 2024.

